# Brain health operationalized as brain parenchymal fraction impacts stroke severity both in acute phase and 3 months post stroke

**DOI:** 10.1093/braincomms/fcaf360

**Published:** 2025-09-16

**Authors:** Roza M Umarova, Laura Gallucci, Arsany Hakim, Marcel Arnold, Christoph Sperber

**Affiliations:** Department of Neurology, Inselspital, University Hospital Bern, University of Bern, Bern 3010, Switzerland; Department of Neurology, Inselspital, University Hospital Bern, University of Bern, Bern 3010, Switzerland; Department of Neuroradiology, Inselspital, University Hospital Bern, University of Bern, Bern 3010, Switzerland; Department of Neurology, Inselspital, University Hospital Bern, University of Bern, Bern 3010, Switzerland; Department of Neurology, Inselspital, University Hospital Bern, University of Bern, Bern 3010, Switzerland

**Keywords:** brain health, prediction, stroke outcome, brain atrophy, brain parenchymal fraction

## Abstract

Stroke lesion imaging alone is not sufficient to predict stroke severity and outcome at a clinically meaningful level, and non-lesional factors are to be defined to enable stroke prognosis and individually tailored therapy. While brain health concept is mainly discussed in the context of primary prevention of neurological diseases, quantitative parameters of brain health like brain parenchymal fraction (BPF) might be associated with better maintenance of function and compensation in (acute) brain pathology. We aimed to investigate whether BPF independently mediates neurological impairment and functional outcome after stroke. We retrospectively analysed patients with first-ever middle cerebral artery stroke. We used generalized linear models with gamma distribution and log link to model neurological impairment [NIH Stroke Scale (NIHSS) 24 h and 3 months] and ordinal logistic regression to model functional outcome at 3 months (modified Rankin scale, 0–6) with the independent variables age, sex, BPF and lesion size. We analysed data of 832 patients (mean age: 67.7 ± 15.3 years, female: 43.5%, median NIHSS 24 h: 3 (1–6)]. A higher BPF was associated with lower neurological impairment: 10% higher BPF was associated with a 16% reduction of NIHSS 24 h (mean ratio 0.840, 95% confidence interval [CI] 0.751–0.940) and a 15% reduction of NIHSS 3 months (mean ratio 0.845, 95% CI 0.745–0.957) independent of age, sex and lesion size. Similarly, BPF had an independent protective effect on functional disability 3 months post stroke (10% higher BPF decreased the odds of worse outcome for 1 modified Rankin scale point by odds ratio [OR] 0.593; 95% CI 0.407–0.864). Brain health, operationalized as BPF, is independently associated with lower neurological impairment both 24 h and 3 months post stroke and better functional stroke outcome. BPF might improve the prediction of stroke outcome and explain interindividual variability in lesion–outcome associations: the same lesion load leads to higher neurological impairment in the presence of brain atrophy, whereas the lesion burden might be clinically less apparent in healthier brains. The data might improve personalized treatment prospects and suggest that efforts on brain health improvement might represent both the primary and secondary reduction of burden of stroke.

## Teaser

Umarova *et al*. report that greater brain parenchymal fraction, representing better brain health, is associated with less neurological impairment in acute stroke and better functional outcome 3 months post stroke, independent of age, sex and lesion size. The findings suggest that overall brain health reduces the individual burden of stroke and may improve individual outcome prediction.

## Graphical Abstract

**Graphical Abstract:**
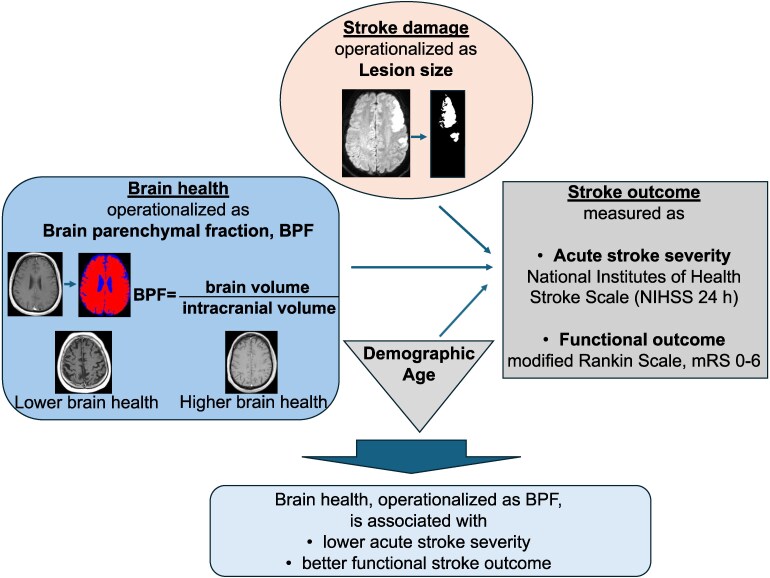

## Introduction

The burden of stroke remains high despite progress in diagnostics and treatment of this acute condition.^1^ Individualized therapeutic approaches are expected to improve stroke outcome but require further improvement of the individual stroke prognosis.^2^ High-dimensional prognostic algorithms on stroke imaging markers were proposed for this aim.^3,4^ However, lesion factors explain far less than half of the variance in stroke outcome,^5^ and further parameters influencing stroke outcome are to be identified.

Several concepts have been introduced to describe the brain's ability to maintain function and recover in the face of brain pathology. They include several related but not equivalent terms such as brain reserve, brain health, brain frailty or resilience and brain age. There are no simple or obvious variables to represent any of these concepts, hence their operationalization remains challenging.^6^ For example, the broader spectrum of brain health encompasses stroke, dementia, vascular cognitive impairment, cognitive ageing, age-related memory loss, vascular functional impairment and subclinical vascular diseases including white matter hyperintensities, brain atrophy, silent brain infarctions and cerebral microbleeds.^7,8^ While further efforts are required for their operationalization and application in individualized patient care, the pragmatic way might be the assessment of ‘structural’ or ‘brain’ reserve representing quantitative brain characteristics at the time of a stroke.^6,9^ Analogous to neurodegenerative diseases,^9^ these parameters may also be applicable to describe the brain’s ability to adapt and compensate for the focal damage in stroke and explain interindividual variability in outcomes.^10^ Specifically, the more the brain structure is available, the lower is the individual lesion load and the easier is the maintenance of a function.^6,11^ In several studies, brain health was represented through multidimensional composed variables.^12,13^ In stroke research, brain health is often represented on a low-dimensional level as brain atrophy or white matter hyperintensities.^14-16^ Brain atrophy might be measured using brain parenchymal fraction (BPF), which reflects the proportion of brain tissue in the total intracranial volume and represents a surrogate measure of brain atrophy.^17^ This parameter decreases along the lifespan, correlates strongly with demographic and biological age and represents the severity of atrophy associated with normal and pathological ageing^18,19^ reflecting the individual’s brain age and health.^20,21^

BPF was previously shown to be associated with functional stroke outcome measured as functional disability 3 months post stroke.^22^ Other measures of brain atrophy were also shown to be negatively associated with functional stroke outcome.^23,24^ However, brain atrophy is strongly associated with frailty, pathological ageing and multimorbidity.^19,25,26^ Previous studies did not control for pre-stroke disability; therefore, it remains unclear whether the negative association between brain atrophy and stroke outcome was not driven by pre-stroke disability. Moreover, it has yet to be determined whether BPF or brain atrophy also modifies the impact of a stroke lesion on stroke severity, thereby influencing lesion-functional correlates. Brain health measures such as BPF might reflect the brain’s ability to respond to brain pathology, maintain function and compensate for the lesion.^10^ Herein, we hypothesized that higher BPF is associated with lower neurological impairment and better functional outcome independent of pre-stroke frailty.

## Methods

### Patients and clinical assessment

In this monocentric retrospective observational study, we included patients treated for acute ischaemic stroke at the Inselspital Bern Stroke Unit between January 2015 and October 2020. Patients with first-ever middle cerebral artery ischaemic stroke confirmed by MRI were eligible. Exclusion criteria were (i) no MRI acquired about 24 h after stroke onset, (ii) additional infarcts outside the anterior circulation and (iii) recurrent stroke within 3 months post stroke, as it would hamper the interpretation of lesion-functional correlates for outcome measures 3 months post stroke (Supplementary Fig. 1). Written institutional general consent was obtained from all participants or their guardians, and the study received approval from the local ethical committee (Kantonale Ethikkommission Bern KEK 2020-02273).

As stroke outcome measure, we used stroke severity in the acute stroke phase measured using NIH Stroke Scale (NIHSS) 24 h after stroke onset, which was routinely assessed during the hospitalization. We used NIHSS 24 h as it better reflects the final lesion load and stroke severity after reperfusion therapy than the NIHSS at admission, which represents the penumbra and the infarct core and does not reflect the application and success of reperfusion therapy. Functional outcome 3 months post stroke was assessed using the modified Rankin scale (mRS) ranging from 0 (=no disability) to 6 (=death) during clinical follow-up in person or by telephone. In the analysis of the association of BPF with stroke disability, we excluded patients for whom any pre-stroke disability (mRS > 0) was recorded. In addition, in part of the patients, NIHSS 3 months post stroke was not available.

### Brain imaging and calculation of BPF

We analysed clinical routine MRI acquired at admission or ∼24 h after symptom onset. MRI examinations were performed on either a 1.5  or 3 T scanner (Magnetom Avanto, Aera, Skyra, Verio, or Vida, Siemens Medical Solutions, Erlangen, Germany). The following MRI sequences from the emergency stroke protocol were used for the analysis: axial diffusion-weighted imaging with apparent diffusion coefficient (4–5 mm slice thickness), fluid-attenuated inversion recovery (4–5 mm slice thickness) and axial T_1_-weighted image (4–5 mm slice thickness). Patients with pronounced artefacts in the MRI images were excluded from the analysis.

We applied a semiautomatic stroke lesion segmentation using diffusion-weighted MRI scans acquired ∼24 h post stroke and a region-of-interest toolbox in SPM12 (http://www.fil.ion.ucl.ac.uk/spm/software/spm12), as previously described.^27^ We manually selected intensity thresholds to optimally map the hyperintense diffusion-restricted brain tissue. Other available sequences (e.g. fluid-attenuated inversion recovery and apparent diffusion coefficient maps) were consulted to support lesion delineation. Lesion masks were then normalized to the standard Montreal Neurological Institute (MNI) space at 2 × 2 × 2 mm^3^ resolution with normalization parameters from the coregistered T_1_ scans. The volumes of MNI-normalised lesion maps were calculated (=‘absolute’ lesion size) and used in the main analysis. In addition, we calculated the individual lesion load, which is defined as the ratio of lesion size in native space to brain volume (BV) (=‘relative’ lesion size). We performed a control analysis implementing relative lesion size instead of ‘absolute’ lesion size of MNI-normalized lesion maps.

Patients’ T_1_ scans were segmented into grey and white matter and cerebrospinal fluid in the native space using the segmentation procedure implemented in SPM12. Since we used T_1_ images acquired in acute stroke, the estimation of BV was not affected by the stroke lesion. Segmentation performance was checked for each patient by RU, and patients with failed segmentation were excluded from further analysis. Next, a BV mask was created as the sum of grey and white matter segments followed by the binarization at a probability of >0.2. Similarly, the cerebrospinal fluid segment was binarized at a probability of >0.2. The volumes of brain masks and cerebrospinal fluid segments were calculated. BPF was calculated based on the measures for the BV mask and cerebrospinal fluid volume (CBF) according to the formula BPF=BV/(BV+CBF).^17^

### Statistical analyses

The distribution of stroke outcome measures (NIHSS 24 h, NIHSS 3 months and mRS 3 months) and lesion size was strongly left-skewed. Therefore, we used generalized linear models with a gamma distribution and a log link for continuous NIHSS scores and ordinal proportional odds logistic regression for mRS measures (0–6). As independent predictors, we introduced age, lesion size, sex and BPF. For mRS 3 months, patients with records of pre-stroke disability (pre-stroke mRS > 0) were excluded from the analysis. Correlation analyses were performed using non-parametric Kendall’s tau tests. Only complete datasets were used and no data were imputed; therefore, the sample size differed across analyses of NIHSS 24 h, NIHSS 3 months and mRS 3 month. We did not included stroke therapy in the statistical models, as the application of reperfusion therapy and its success are reflected in the final lesion size 24 h post stroke.

Given the strong correlation between chronological age and BPF,^18^ we performed a sensitivity analysis to verify that potential effects of BPF are not driven by the covariate age. Since a mediation analysis with ordinal target variables is not widespread and difficult to interpret, we refrained from this and chose an alternative approach that is easy to interpret. We first ran a linear regression with age as a predictor and BPF as the dependent variable and computed standardized residuals representing BPF variability not explained by age. Second, we used these residuals as a predictor in another regression analysis that mirrored the main analysis. For this analysis, all other input variables (age and lesion size) had to be also standardized. Another sensitivity analysis included the individual lesion load instead of absolute size of MNI-normalised lesions. All statistical tests were performed at *α* = 0.05. IBM SPSS statistics 28.0.1.1 was used.

## Results

The demographic data of patients included in the study are reported in Table 1. The four predictor variables age, sex, lesion volume and BPF were correlated. We observed (i) a moderate correlation between age and BPF (Kendall’s *τ*_b_ = −0.473; *P* < 0.001) showing that BPF decreased with increasing age, (ii) a weak correlation between lesion size and BPF (*τ*_b_ = 0.110; *P* < 0.001) indicating that patients with healthier brains had larger lesions, (iii) a weak correlation between sex and age (*τ*_b_ = 0.142; *P* = 0.001) indicating that female patients were older in the sample and (iv) a weak correlation between lesion size and age (*τ*_b_ = − 0.071; *P* < 0.001) indicating that older patients had smaller lesions. The distribution of BPF did not significantly deviate from normality (Lilliefors test: *P* = 0.50; *n* = 832). Supplementary Fig. 2 provides a lesion overlap of the study sample.

**Table 1:** Demographic and clinical data of patients included in the main analysis of acute stroke severity

Clinical and radiological variables	All participants (*N* = 832)
Age, years median (IQR; range)	69.8 (58.6, 79.6; 16.7–97.3)
Sex, male %	56.5
Pre-stroke mRS, median (IQR; range)	0 (0, 0; 0–5)
NIHSS at admission, median (IQR; range)	6 (3, 12; 0–36)
NIHSS 24 h, median (IQR; range)	3 (1, 6; 0–36)
Therapy, no/IVT/MT/both, %	19.5/28.1/29.9/22.5
Lesion lateralization, left/right/bilateral	440/372/20
3 months mRS, median (IQR; range)	1 (0, 2; 0–6)
Lesion size median (IQR; range)	
Individual lesion load^a^, %	1.04 (0.26, 3.04; 0.002–26.67)
Lesion size (MNI space), ml	18.1 (4.5, 51.7; 0.1–397.4)
Brain parenchymal fraction, median (IQR; range), %	72 (68, 76; 57–90)
Large vessel occlusion, %	65.5
History of TIA, %	4.6
Hypertension, %	67.2
Diabetes mellitus, %	15.8
Smoking, %	26.8
Hyperlipidaemia, %	68.7
Atrial fibrillation, %	26.5
Coronary heart disease, %	16.6
Body mass index, median (IQR; range)	25.9 (23.7, 29.1; 15.1–48.4)

MT, mechanical thrombectomy; IQR, interquartile range; IVT, intravenous thrombolysis; TIA, transient ischaemic attack.

^a^Individual lesion load is calculated as lesion size in native space divided by BV multiplied by 100%.

### Association between BPF and stroke outcome measures

The regression analysis for NIHSS 24 h (generalized linear model gamma log link) demonstrated that higher BPF was positively associated with lower stroke severity in the acute stroke phase: a 10% higher BPF led to a 16% reduction of NIHSS 24 h (mean ratio 0.840; 95% CI 0.751–0.940; Table 2) independent of age, lesion size and sex. Each 10 years older age were associated with a 7.5% higher NIHSS 24 h (mean ratio 1.075; 95% CI 1.030–1.122).

**Table 2:** Results of regression analysis on association of BPF with stroke severity 24 h and 3 months post stroke and ordinal logistic regression for functional stroke outcome at 3 months (mRS, 0–6)

Outcome measure	Predictors	Mean ratio	95% CI	*P*
NIHSS 24 h, *n* = 832	Intercept	**6**.**735**	**2**.**449–18**.**519**	**0**.**000**
	BPF, per 10% change	**0**.**840**	**0**.**751–0**.**940**	**0**.**002**
	Sex, female	1.022	0.927–1.126	0.665
	Age, per 10 years	**1**.**075**	**1**.**030–1**.**112**	**<0**.**001**
	Infarct size, ml	**1**.**009**	**1**.**008–1**.**010**	**<0**.**001**
NIHSS 3 months, *n* = 464	Intercept	3.104	0.979–9.836	0.054
	BPF, per 10% change	**0**.**845**	**0**.**745–0**.**957**	**0**.**008**
	Sex, female	1.957	0.860–1.064	0.417
	Age, per 10 years	**1**.**065**	**1**.**017–1**.**116**	**0**.**007**
	Infarct size, ml	**1**.**007**	**1**.**006–1**.**008**	**<0**.**001**
mRS 3 months, *n* = 602	BPF, per 10% change	**0**.**593**	**0**.**407–0**.**864**	**0**.**007**
	Sex, female	1.139	0.848–1.529	0.389
	Age, per 10 years	1.059	0.929–1.206	0.392
	Infarct size, ml	**1**.**014**	**0**.**012–1**.**017**	**<0**.**001**

Significant results are highlighted in bold.

For NIHSS 3 months (*n* = 464), a 10% higher BPF was associated with a 15% reduction of stroke severity (0.845; 95% CI 0.745–0.957) independent of age, lesion size and sex. Each 10 years older age were associated with a 6% higher NIHSS 3 months (1.065; 95% CI 1.017–1.116).

Similarly, BPF was associated with better functional stroke outcome 3 months post stroke measured as mRS (0–6) (generalized linear model ordinal logistic regression, only patients without records of pre-stroke disability were included, *n* = 602): a 10% higher BPF decreased the odds of worse outcome for 1 mRS point by a factor of 0.593 (95% CI 0.407–0.864), independent of age, lesion size and sex.

### Sensitivity analysis

Given the negative correlation between age and BPF (Supplementary Fig. 3), as well as the known negative impact of age on stroke outcome, we performed a sensitivity analysis. To this aim, we repeated the previous analysis with standardized age-adjusted BPF residuals instead of original BPF values; all other predictors were the same as in the main analysis. The results were confirmed: age-adjusted BPF was associated with lower neurological impairment both 24 h and 3 months post stroke as well as with lower functional disability 3 months post stroke (Supplementary Table 1). Similarly, the inclusion of individual lesion load in the models instead of absolute size of MNI-normalised lesions did not change the results (Supplementary Table 2).

## Discussion

We investigated the relationship between BPF as a proxy for brain health and stroke outcome measures in the acute phase and 3 months post stroke whereby BPF was associated with lower stroke severity and more favourable functional stroke outcome independent of age, sex and lesion volume. Even though BPF correlated with the baseline predictor age, control analyses confirmed the independent impact of BPF beyond its relation to demographic age. The effect of brain health or reserve on the functional manifestation of brain pathology was suggested and described for cognitive functions.^9^ We demonstrated its protective impact on the general level of post-stroke neurological impairment even in the acute stroke stage, as measured by the NIHSS score.

BPF serves as an individual surrogate marker of brain changes associated with normal and pathological aging^19,25,26^ and can act as a proxy for overall brain health. BPF provides insight into the accumulated damage sustained by the brain over the course of a lifetime. It can be easily extracted from routine clinical MRI scans, making it readily available for use in clinical practice. BPF was related to a slower rate of memory decline in ageing.^28^ It might impact the effectiveness of rehabilitation after stroke.^29^ BPF predicted disability and cognitive status in Cerebral Autosomal Dominant Arteriopathy with Subcortical Infarcts and Leukoencephalopathy (CADASIL)^30,31^—a genetic small vessel disease with neurodegenerative changes. It was shown to relate to disease progression and neuronal loss in multiple sclerosis.^32^ BPF was related to other pathological parameters such as glycated haemoglobin, white matter hyperintensities,^19^ accruing Alzheimer disease and vascular pathology,^21^ and a history of traumatic brain injury.^33^ Both previous studies^22-24^ and the current one controlling for pre-stroke disability and previous strokes provide evidence for the impact of BPF on functional stroke outcome. Data on the association between BPF and acute stroke severity were missing until now.

Several mechanisms might explain the protective impact of quantitative parameters of brain health or brain reserve on stroke outcome.^11^ Better brain health might mitigate the functional impact of stroke lesion, first, by representing more neural resources: the more structure available, the less impact a stroke of specific size and location has.^9^ Second, brain health might influence neural mechanisms of maintenance and plasticity^6^ and consequently stroke recovery. While stroke leads to widespread recovery-relevant remodelling,^34^ brain health might influence the brain plasticity required for this remodelling by providing a reliable and efficient neuronal backup architecture. Lower BPF might be related to neuropathological changes^21^ and, therefore, indicate less healthy brain tissue available for the recovery process than in patients with higher BPF. In line with this, brain atrophy was associated with worse stroke recovery,^23^ whereas higher interhemispheric white matter integrity correlated with better motor recovery.^35^ Finally, brain health or reserve might impact a network's compensation by enhancing the recruitment of potentially still available secondary neural resources.

Our study showed that lesion-functional associations are modulated by brain health parameters already in acute stroke, i.e. an individual’s brain health impacts stroke outcome independent of lesion size and age. Hence, the lesion load might be overestimated in less healthy brains and underestimated in healthier brains. Furthermore, a higher BPF and, thereby, a healthier brain, were associated with a larger lesion volume in the present study. In light of the present finding, this correlation can potentially be explained by the following reasons: (i) small strokes might be more likely to be silent in a healthy brain, (ii) patients with low brain health might be frailer and more often die from larger lesions and (iii) small lesions might represent a lacunar stroke as a feature of small vessel disease and thus of low brain health or reserve itself. Consequently, the correlation between BPF and lesion size might mask a possible protective effect of brain health: a less healthy brain might tolerate less pathology and even small ischaemic lesions may become functionally apparent. Given that triage and management of stroke patients depend on the severity of neurological deficits measured by NIHSS, important discussion points arise here: do we sometimes under-treat and under-rehabilitate patients with healthier brains? Should our triage and therapy be guided by the severity of neurological impairment, lesion size or both? Patients with a healthier brain cope better with lesions and demonstrate less neurological impairment and better functional outcome, but their brain is still damaged to a higher extent than reflected by neurological scales. However, not undergoing treatment for a stroke could then result in long-term consequences in functional or cognitive outcome, e.g. by increasing the risk of dementia.^36^

One of the study limitations is the use of T_1_ scans that have been acquired for clinical routine and do not include high-resolution scans. MRI examination was performed at different scanners that impacted the comparability of voxel classification into grey and white matter segments. However, this could not have significantly biased the results as we operated with the sum of grey and white matter segments and not with grey or white matter segments separately. In addition, there are many calls in the research community for the greater integration of clinical routine data into research for the generation of big datasets. The present study is in line with the calls for sustainability in research. Another study limitation is the inclusion of only patients with anterior circulation stroke. Conversely, posterior circulation stroke has distinct lesion-functional correlates and functional outcomes. Consequently, this patient group is often investigated separately from anterior circulation stroke. In addition to the issue of generalizability, it is important to consider the possibility that the reported effect size may be quantitatively specific to the population and data distribution of the present study. Nevertheless, the generalizability of the reported associations is ensured by the use of a representative sample size, a reliable and valid statistical model and sensitivity analyses. Next, though we used lesion size instead of lesion location in the analysis, the former is still a comparably informative biomarker for stroke damage in an anterior circulation stroke population,^5^ as in the present study. In addition, we focused only on one quantitative proxy of brain health and did not include other measures, e.g. white matter hyperintensities or severity of frontal or hippocampus atrophy. However, these parameters were the focus of previous research,^37^ and future studies might investigate all these parameters multidimensionally.

Altogether, the present results help us to understand interindividual variability in stroke patients and suggest the consideration of quantitative proxies of brain health in large trials.^11^ The data might help improve the prediction of (long-term) stroke outcome. The effect of brain health on the functional manifestation of stroke, analogous to the brain reserve concept formulated for neurodegenerative disease, suggests a potential knowledge transfer from research on neurodegeneration. Furthermore, the results draw possible synergy effects in preventive interventions in neurodegeneration, stroke and healthy ageing.^38^ They underline the global impact of healthy and disease-modifying lifestyle to improve brain health for primary and secondary (outcome improvement) stroke prevention.^11,39,40^

## Supplementary Material

fcaf360_Supplementary_Data

## Data Availability

Clinical data are not publicly available, but qualified researchers may request the corresponding author for access to anonymised data. Proposals need to be approved by the local ethics committee.
