# Supplementary material for: Brain health operationalized as brain parenchymal fraction impacts stroke severity both in acute phase and 3 months post stroke
Source: Brain Commun. 2025 Sep 16;7(5):fcaf360. doi: 10.1093/braincomms/fcaf360 (PMC12495403; doi:10.1093/braincomms/fcaf360)
Supplement: fcaf360_Supplementary_Data [file fcaf360_supplementary_data.docx]

**Supplementary material**

Supplementary Table 1. Control regression analysis with age-adjusted BPF residuals.

| Outcome measure | Predictors | Mean ratio | 95% CI | | *p* |
| --- | --- | --- | --- | --- | --- |
| NIHSS 24 h, n=832 | Intercept | **0.841** | **0.792** | **0.893** | **<0.001** |
|  | BPF | **0.931** | **0.889** | **0.975** | **0.002** |
|  | Sex, female | 1.021 | 0.931 | 1.119 | 0.662 |
|  | Age | **1.186** | **1.130** | **1.243** | **<0.001** |
|  | Infarct size | **1.633** | **1.551** | **1.719** | **<0.001** |
| NIHSS 3 months, n=464 | Intercept | **0.936** | **0.894** | **0.980** | **0.005** |
|  | BPF | **0.958** | **0.924** | **0.993** | **0.018** |
|  | Sex, female | 0.972 | 0.906 | 1.043 | 0.428 |
|  | Age | **1.105** | **1.065** | **1.146** | **<0.001** |
|  | Infarct size, ml | **1.328** | **1.272** | **1.386** | **<0.001** |
| mRS 3 months, n=602 | BPF | **0.798** | **0.679** | **0.939** | **0.007** |
|  | Sex, female | 1.139 | 0.848 | 1.529 | 0.389 |
|  | Age | **1.339** | **0.152** | **1.557** | **<0.001** |
|  | Infarct size, ml | **2.312** | **1.981** | **2.697** | **<0.001** |

Please, note that due to the adjusting procedure for age followed by standardisation, the direct comparison of size effects from the main analysis is not possible.

Supplementary Table 2. Control regression analysis including individual lesion load instead of absolute size of MNI-normalised stroke lesions.

| Outcome measure | Predictors | Mean ratio | 95% CI | | *p* |
| --- | --- | --- | --- | --- | --- |
| NIHSS 24 h, n=832 | Intercept | **6.249** | **2.260** | **17.276** | **0.000** |
|  | BPF, per 10% change | **0.854** | **0.763** | **0.956** | **0.006** |
|  | Sex, female | 1.035 | 0.939 | 1.141 | 0.487 |
|  | Age, per 10 years | **1.071** | **1.026** | **1.118** | **0.002** |
|  | Individual lesion load, % | **1.147** | **1.113** | **1.164** | **<0.001** |
| NIHSS 3 months, n=464 | Intercept | 3.452 | 1.142 | 10.438 | 0.028 |
|  | BPF, per 10% change | **0.852** | **0.753** | **0.965** | **0.011** |
|  | Sex, female | 0.964 | 0.867 | 1.071 | 0.491 |
|  | Age, per 10 years | **1.062** | **1.015** | **1.111** | **0.010** |
|  | Individual lesion load, % | **1.116** | **0.096** | **1.135** | **<0.001** |
| mRS 3 months, n=602 | BPF, per 10% change | **0.617** | **0.424** | **0.899** | **0.012** |
|  | Sex, female | 1.163 | 0.866 | 1.561 | 0.317 |
|  | Age, per 10 years | 1.062 | 0.932 | 1.210 | 0.365 |
|  | Individual lesion load, % | **1.257** | **1.206** | **1.310** | **<0.001** |

Supplementary Figure 1. Study flowchart.

**
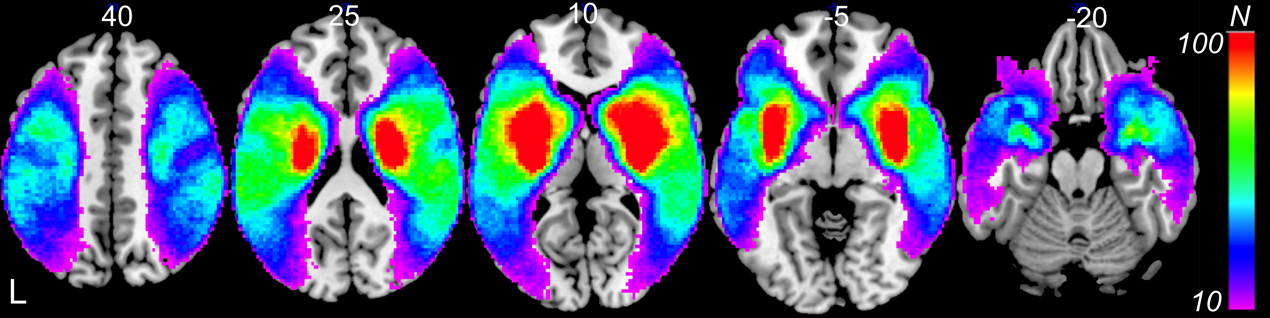
**

Supplementary Figure 2. Stroke lesion overlap of the study sample. Z-coordinates of MNI-space are provided. Colour bar indicates the number of overlapping lesions.


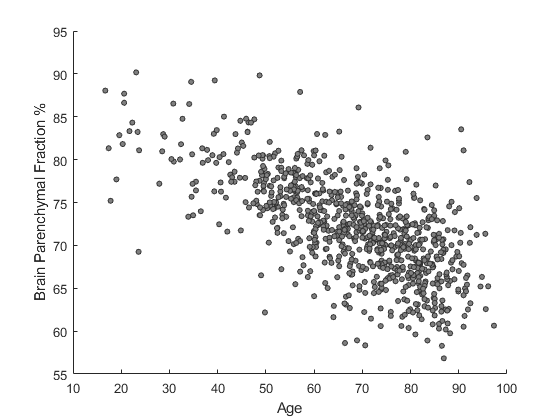


Supplementary Figure 3. Relationship between the demographic age and brain parenchymal fraction. Scatter plot between demographic age (x-axis) and brain parenchymal fraction (y-axis) is provided; each point represents a single patient.
